# Supervised injection facilities in Canada: past, present, and future

**DOI:** 10.1186/s12954-017-0154-1

**Published:** 2017-05-18

**Authors:** Thomas Kerr, Sanjana Mitra, Mary Clare Kennedy, Ryan McNeil

**Affiliations:** 1 British Columbia Centre on Substance Use, St. Paul’s Hospital, 608-1081 Burrard Street, Vancouver, B.C. V6Z 1Y6 Canada; 2 Department of Medicine, University of British Columbia, St. Paul’s Hospital, 608-1081 Burrard Street, Vancouver, BC V6Z 1Y6 Canada; 3 Ontario HIV Treatment Network, 1300 Yonge Street, Suite 600, Toronto, ON M4T 1X3 Canada; 4 School of Population and Public Health, University of British Columbia, 5804 Fairview Avenue, Vancouver, BC V6T 1Z3 Canada

**Keywords:** Supervised injection facilities in Canada

## Abstract

Canada has long contended with harms arising from injection drug use. In response to epidemics of HIV infection and overdose in Vancouver in the mid-1990s, a range of actors advocated for the creation of supervised injection facilities (SIFs), and after several unsanctioned SIFs operated briefly and closed, Canada’s first sanctioned SIF opened in 2003. However, while a large body of evidence highlights the successes of this SIF in reducing the health and social harms associated with injection drug use, extraordinary efforts were needed to preserve it, and continued activism by local people who inject drugs (PWID) and healthcare providers was needed to promote further innovation and address gaps in SIF service delivery. A growing acceptance of SIFs and increasing concern about overdose have since prompted a rapid escalation in efforts to establish SIFs in cities across Canada. While much progress has been made in that regard, there is a pressing need to create a more enabling environment for SIFs through amendment of federal legislation. Further innovation in SIF programming should also be encouraged through the creation of SIFs that accommodate assisted injecting, the inhalation of drugs. As well, peer-run, mobile, and hospital-based SIFs also constitute next steps needed to optimize the impact of this form of harm reduction intervention.

## Background

Canada has long contended with health-related and social harms associated with injection drug use. In response, municipalities throughout the country have implemented a range of harm reduction policies and programs. However, support for harm reduction approaches in Canada has been mixed and contested in various arenas, including in the country’s highest court [1].

In the mid-late 1990s, drug-related harms peaked in the city of Vancouver. With an annual incidence of HIV infection of 19% among local people who inject drugs (PWID), and over 300 fatal overdoses occurring in the province of British Columbia, Vancouver’s health authority declared a public health emergency [2]. A series of events and actions that followed eventually led to the opening of Canada’s first unsanctioned and sanctioned supervised injection facilities (SIFs) [3]. As in other SIFs internationally, PWID can inject pre-obtained drugs under nurse supervision at Vancouver’s sanctioned SIFs, as well as access sterile injection equipment, receive emergency overdose response and referrals to a range of internal and external programs [4]. Since this time, efforts to establish SIFs have persisted, numerous studies have demonstrated the health and social benefits of SIFs, innovations in SIF delivery have occurred, and new SIFs are now being implemented throughout the country. Herein, we review the experience with SIFs in Canada, with a focus on the past, present, and future.

### SIFs in Vancouver: early history

In response to the provincial overdose crisis, in 1994, the Provincial Chief Coroner of British Columbia formed a task group that produced the “Cain Report” [3]. Among their recommendations was that Vancouver explore SIFs given the experience with these facilities in Europe [3]. However, no immediate plans were initiated by local health authorities to implement SIFs.

In the wake of the Cain Report, interest in SIFs grew in Vancouver, particularly among local PWID. In 1995, a peer-led group, IV Feed, opened and operated an unsanctioned drug user-run SIF known as the Back Alley with the support of local activists Ann Livingston and Bud Osborn [5]. Sign-in sheets collected at the site indicated that the Back Alley SIF accommodated over 100 PWID each night, and accounts suggest that local street nurses visited the site to provide support [5]. Although some PWID reported having been referred to the IV Feed site by police officers, the site was closed by police after approximately 1 year of operation [5].

Efforts to establish SIFs in Vancouver were further bolstered in 2000–2001 when the City of Vancouver released its Four Pillar Drug Strategy, which was based on policy models from Western Europe attempting to balance prevention, enforcement, treatment, and harm reduction [6]. Included in the strategy was a call for two SIFs. Although City Council endorsed the strategy in 2001 [5], the challenge remained that the City did not have responsibility for implementing health programs, as provinces are responsible for healthcare administration in Canada. Still, the Four Pillar Strategy served to further ignite public dialog and education about drug-related harm and the potential of SIFs as one part of a larger strategy.

Around the same time, a number of public events focused on drug use led to increased interest in SIFs. These events included visits from European officials with experience with SIFs [5] and prompted one group, known as the Harm Reduction Action Society, to develop a full proposal for a pilot SIF [7]. This group included a range of stakeholders, including local PWID, activists, healthcare professionals, researchers, and families of people who use drugs. SIFs soon after became an issue during a municipal election, with every party stating that they would implement SIFs if elected. Larry Campbell, a former Royal Canadian Mounted Police Officer, was elected mayor in 2002 and promised to establish a SIF within a month of being elected [8]. However, prior to a SIF being opened, a large police crackdown was initiated in Vancouver’s Downtown Eastside (DTES). In response, local activists and PWID opened an unsanctioned SIF to protest the crackdown and delays in opening a SIF [9]. The “327 Carrall Street SIF” operated for 184 days, during which time the SIF volunteers supervised over 3000 injections [10]. This SIF, like many of the other unsanctioned SIFs before it, was eventually closed due to pressure from local police and policy makers [10].

In 2002, another important development occurred when nurses working at the Dr. Peter Centre began supervising injections [11]. The Dr. Peter Centre operates a day program and a residence for people living with HIV/AIDS. These activities of the Dr. Peter Centre were eventually made public following a series of consultations with the provincial professional nursing association, the Registered Nursing Association of British Columbia (RNABC) [11]. Representatives from RNABC informed the Dr. Peter Centre nurses that, in the opinion of the association, the supervision of injections fell within the scope of acceptable nursing practice even if those injections were of illegal substances [11]. The RNABC also went one step further and indicated that the supervision of injections in a setting like the Dr. Peter Centre was part of the nurses’ ethical obligations given the potential harms that could arise from unsupervised injections [11].

### Insite: Canada’s first sanctioned SIF

In September 2003, Canada’s first legally sanctioned SIF opened. This came about after a Vancouver-based non-government organization, the Portland Hotel Society (PHS), quietly built a SIF within a boarded up and seemingly vacant building, and then one day announced publicly that the SIF had been built [3]. Eventually, the regional health authority agreed to work with the PHS to open the SIF, although it is unclear how long this may have taken if the PHS had not taken the rather extraordinary measure of building the physical site in secret.

Health Canada had released its SIF guideline document shortly before this development, which set out how individual municipalities could obtain an exemption from the federal Health Minister to legally establish a SIF [3, 5]. This document laid out numerous conditions and required site visits by Health Canada officials, but eventually Insite opened with federal approval of an exemption under Section 56 of the Controlled Drugs and Substances Act granted by the federal Health Minister. The site includes 13 spaces for injecting and is usually open 18 h a day from 10 am to 4 am [12], although some experimenting with 24 h of operation has been undertaken.

Insite was opened under the condition that it operate as a scientific pilot and be rigorously evaluated. This was deemed essential, especially given the limited peer-reviewed data specific to SIFs in Europe. The evaluation quickly showed that Insite was meeting its objectives of reducing public disorder [13], infectious disease transmission [14, 15], and overdose [16] and was successfully referring individuals to a range of external programs, including detoxification and addiction treatment programs [17, 18]. Further, the evidence indicated that Insite was not resulting in increases in crime or promoting initiation into injecting [19, 20], and Insite was found to be cost-effective [15, 21]. To date, over 40 peer-reviewed studies have been published which speak to the many benefits and lack of negative impacts of this site.

Despite the success of Insite, the facility came under fire from many sides. Importantly, in 2006, in the final year of the three-year pilot study of Insite, Canada elected a new Conservative government, which was publicly vocal in its opposition to harm reduction and Insite in particular [22]. Coinciding with this, a number of groups in Canada and internationally, including the Drug Free America Foundation, The Drug Prevention Network of Canada, and Drug Free Australia, began politicizing and misrepresenting the evidence generated from the evaluation of Insite [22, 23]. This culminated in Drug Free Australia submitting a complaint to the researchers’ university alleging the team had engaged in academic misconduct and falsified data. An arms-length investigation was undertaken, and the complaint was quickly dismissed as having no merit [24].

Eventually, the PHS and two local drug users (Dean Wilson and Shelly Tomic) took the federal government to the Supreme Court of British Columbia in an effort to prevent the closure of Insite [25]. The Supreme Court judge ruled in support of the continued operation of Insite, recognized it as a health service, and noted that it would be unconstitutional to deny PWID access to this life saving service [25]. The federal government appealed, and the appeal court judges also ruled in favor of the continued operation of Insite [26]. Again, the federal government appealed to the Supreme Court of Canada [27]. The PHS and local PWID leading the case were supported by a range of intervenors, including the Canadian Medical Association, the Canadian Association of Nurses, and the Canadian Public Health Association [27]. The Supreme Court justices ruled 9–0 in favor of the continued operation of Insite and in their decision stated:

“The Minister’s failure to grant [an exemption] to Insite…contravened the principles of fundamental justice…Insite has been proven to save lives with no discernable negative impact on the public safety and health objectives of Canada…(p. 139)” [27]

The federal government was then granted 1 year to revise its policies to allow for the legal operation of SIFs in Canada. The government responded with a new bill (Bill C-2), which made opening a SIF more difficult than before, listing 26 conditions that had to be met before a SIF could be opened [28]. Among them, municipalities had to have local community support and the support of local police. This led many, including the Canadian Medical Association, to criticize the new bill [28], although the majority government was able to pass it.

While Insite has continued to operate, local evidence suggests that there is much unmet need for SIF services in Vancouver. Although reliable estimates of the size of the PWID population in Vancouver are lacking at this time, several indicators point to the need for further SIFs in this setting. For example, it has been estimated that between 30 and 40 PWID leave Insite each day without accessing the injecting room due to long wait times [29]. Other research has pointed to distance as a primary barrier to accessing Insite [30]. Further, it has been shown that some individuals avoid the block where Insite is located due to past experiences of violence in the immediate vicinity [31].

### SIFs in Montreal, Toronto, Ottawa, and Victoria

Given the ongoing problems with injection drug use throughout Canada and the experience with Insite, a number of other municipalities across Canada began undertaking SIF feasibility research and developing plans for establishing SIFs; included were the cities of Montreal, Toronto, Ottawa, and Victoria [32–36]. Consistent with work done elsewhere, SIF feasibility research suggested that local PWID would use a SIF, although work done in Toronto suggested that, because the population of PWID was spread throughout the city, a greater number of smaller SIFs should be implemented [33]. Further, a cost effectiveness analysis recommended that three SIFs be established in Toronto and two be established in Ottawa [37]. A qualitative evaluation of key stakeholder opinions in Toronto and Ottawa also identified opposition to or concern about SIFs [38]. Specifically, seven reasons for ambivalence were identified, including: “lack knowledge of evidence about SIFs; concern that SIF goals are too narrow…; uncertainty that the community drug problem is large enough to warrant a SIF(s); the need to know more about the “right” places to locate a SIF(s) to avoid damaging communities or businesses; worry that a SIF(s) will renew problems that existed prior to gentrification; concern that resources for drug use prevention and treatment efforts will be diverted to pay for a SIF(s); and concern that SIF implementation must include evaluation, community consultation, and an explicit commitment to discontinue a SIF(s) in the event of adverse outcomes” [38]. Police in Toronto also expressed concerns regarding SIFs [39]. However, follow-up research demonstrated that public opinion regarding SIFs increased over time [32]. As well in Ottawa and Toronto, emphasis was placed on creating integrated supervised injecting services, where PWID could access additional programs and supports [33]. Still, with a Conservative government in power, no sustained efforts were made to create a SIF in these settings.

### Drug user activism

Despite the lack of an enabling environment for SIFs in Canada following the election of a Conservative government, a drug user organization in Vancouver continued to address gaps in service delivery and promote innovation in SIF programming. First, recognizing that the sanctioned SIF did not accommodate people who need assistance with injections due to federal regulations, the Vancouver Area Network of Drug Users (VANDU) began operating a SIF within their offices where people could get manual assistance with injections [40]. An evaluation of this program indicated that VANDU reshaped the social, structural, and spatial contexts of assisted injection practices in a manner that minimized HIV and other health risks, while allowing people who require help injecting to escape drug scene violence [40]. Second, VANDU also operated a safer smoking room for crack users, given that individuals who smoked crack remained vulnerable to arrest and violence when consuming drugs in public [41]. An evaluation of the program demonstrated how a high demand for the safer smoking room was driven by the need to minimize exposure to policing, drug scene violence, and stigma [41]. Further, the program was found to foster harm reduction practices by reshaping the social-structural context of crack smoking and reduced the potential for health harms [41]. The VANDU supervised consumption services were closed after operating for approximately 3 years following a threat from the local health authority to rescind the organization’s funding [42].

### A changing political landscape

In October 2015, Canadians elected a new Liberal government under the leadership of Justin Trudeau, whose government had publicly expressed support for SIFs [43]. Within only a few months of taking office, Health Canada granted a legal exemption to the Dr. Peter Centre [44], after almost 14 years of operation without an exemption. With the change in the political landscape, various municipalities began planning to open SIFs.

The election of the Liberal government coincided with the emergence of opioid overdose epidemics in many places in Canada. This in turn prompted calls for the federal government to amend Bill C-2, which outlined the various conditions that needed to be met in order to open a SIF [45]. After much lobbying, the government introduced a new bill, Bill C-37, to replace Bill C-2 [46]. The new bill replaces the 26 conditions with five conditions, including: demonstration of the need for such a site to exist, demonstration of appropriate consultation of the community, presentation of evidence on whether the site will impact crime in the community, demonstration that regulatory systems are in place, and provision of evidence that appropriate resources are in place [47].

### New SIF feasibility work and planning

With a new government in place that expressed support for SIFs, and given the emergence of the opioid overdose epidemic, a number of municipalities began developing plans to establish SIFs, and several initiated SIF feasibility research. Montreal quickly moved forward with plans to open three SIFs, and in February 2017 obtained approval from the federal government to do so [48]. Polls indicated that public support for SIFs in Montreal was high, and all levels of government were supportive [49]. Plans to open a mobile SIF have also been discussed in Montreal [49]. As well, the City of Vancouver has sought federal approval for several additional SIFs, including a women- only SIF, which is expected to open in 2017 [50]. Other cities with advanced plans to open SIFs included Victoria (one site) [51], Toronto (three sites) [52], Ottawa (one site) [53], Surrey (two sites) [54], and Edmonton (four sites, including a hospital-based site) [55]. However, support for SIFs has varied considerably in these settings. In Ottawa, the mayor and police officials have expressed strong opposition to SIFs, making a number of statements inconsistent with the available evidence, including the suggestion that SIFs increase crime [56, 57]. In Victoria, citizen groups have opposed syringe exchange and other harm reduction programs, and have expressed opposition to SIFs [58]. In Kelowna and Kamloops, public opposition to SIFs, in particular by local business associations [59], appears to have prompted the local health authority to opt for a mobile rather than fixed SIF in each of these municipalities, as gaining acceptance for any specific permanent location for fixed SIFs proved too difficult [60].

A number of other jurisdictions in Canada also began conducting SIF feasibility research in the wake of the change in the federal government. These included small, mid, and more remote municipalities, including in London and Thunder Bay [61–63]. Consistent with results generated elsewhere, these studies found high rates of willingness to use a SIF among local PWID, including among those at high risk for drug-related harms, as well as high levels of key stakeholder support [64]. Discussions about SIF feasibility research and establishing SIFs have occurred in other cities, including Hamilton, Chilliwack, Calgary, and Saskatoon [61, 65–67].

### The fentanyl overdose crisis—overdose prevention sites

Although a number of settings in Canada have been contending with opioid overdose epidemics for some time, the emergence of illicitly manufactured fentanyl—a powerful opioid that has been found in more commonly injected drugs such as heroin—has made the situation worse [68]. For example, the province of British Columbia saw 922 illicit drug overdose deaths in 2016, a 78% increase over 2015, with a growing number of overdoses involving fentanyl [69]. This situation prompted the government of British Columbia to declare a public health emergency [69].

In Vancouver’s DTES, in response to the rapidly increasing number of deaths, local activists including Ann Livingston and Sarah Blythe erected a tent with tables where people could sit and inject or smoke drugs under supervision and receive emergency overdose response as needed [70]. During this time, line ups at the local sanctioned SIF became long and the program was unable to meet demand. The so-called “pop-up safe injection site” was tolerated by local health officials and police, and in time, other pop-up SIFs started to emerge in other settings in the province, including in Nanaimo [71]. This low-threshold SIF model, while not providing the level of or intensity of support offered at Insite, was well-utilized, and many overdoses were reversed at this site [72].

After calls on the federal government to declare an emergency failed, the Health Minister of British Columbia instructed various regional health authorities to open what have become known as “overdose prevention sites” (OPSs) [73]. At these sites, PWID are provided with sterile equipment for injection in a closed indoor setting, and staff (unusually non-nursing staff) provides emergency response in the event of overdose. The motivation for making a distinction between SIFs and OPSs may reflect subtle differences in service design, as well as the ongoing need for federal approval to open a sanctioned SIF and frustration with the time it takes to acquire such approval. Within a couple of days, three new OPSs opened in the DTES, including one in the office of VANDU, which constituted the first sanctioned peer-run model in Canada [74, 75]. These OPSs differ from Insite in several ways. Importantly, the sites are designed primarily to prevent overdoses, do not employ nurses, and offer a lower level of clinical intervention around safer injecting practice and other issues (e.g., diagnosis and treatment of soft tissue infections). The OPSs are also simpler in physical layout, often as a result of their rapid integration into existing spaces rather than implementation within purpose-built facilities. However, sterile injecting supplies are provided, injections are supervised, and naloxone is administered in the event of overdose. As the overdose epidemic continued to rage on, more OPSs opened in Vancouver and throughout the rest of the province, including in Victoria [76]. At this time, there are approximately 18 OPSs operating in the province of British Columbia [73]. The City of Vancouver has also sought federal approval for a women- only SIF, which is expected to open in 2017 [50].

## Discussion

Efforts to establish SIFs in Canada have persisted since the mid-1990s and were undertaken primarily in response to under-addressed epidemics of HIV infection and overdose [5]. Although a sanctioned SIF opened in 2003 [77], the value of SIFs remained contested, leaving Canada’s lone SIF in perpetual pilot status for over a decade. Continued activism by local PWID and nurses led to further innovations in SIF programming [11, 40], changes in the federal government created a more enabling environment, and numerous municipalities have since moved towards opening SIFs. Although progress has been made towards making SIFs a component within the continuum of services offered to PWID, these sites remain difficult to establish, and opportunities to extend this model and promote innovation have been missed.

The opening of Canada’s first sanctioned SIF resulted from the actions taken by a diverse group of community actors (e.g., activists, researchers, health care professionals), including targeted and sustained civil disobedience by PWID and their allies [3, 5]. This experience is consistent with the establishment of harm reduction programming elsewhere in Canada and internationally, as injection drug-using populations and other community actors have circumvented bureaucratic and legal processes to implement innovative programs to reduce social suffering among injection drug-using populations [78–80] Such community organizing and drug user activism proved further necessary to sustain Insite when its operator (PHS Community Services) and two persons who inject drugs (Dean Wilson, Shelly Tomic) preemptively sued to keep the facility open after the then-Conservative federal government appeared positioned to withhold annual exemptions for the facility. As noted, along with the extensive research evidence, this was critical to the ruling that enabled continued operation of Insite.

Despite this landmark ruling, political opposition continues to be the most significant barrier to the expansion of SIFs in Canada [22] Although provinces are responsible for the administration of health care in Canada, the requirement that SIFs receive exemptions to federal drug laws has subjugated local efforts to implement these critical health services to the whims of municipal, provincial, and federal politicians, as successive governments have varied in their positions on SIFs, and the requirement of local support has at times been difficult to obtain. The previous Conservative federal government (2006–2015) opposed their operation and defied the spirit of the Supreme Court of Canada ruling by passing new legislation that erected considerable barriers to their expansion, including the requirement of approval by local police. The more recently elected Liberal government (2015–present) has signaled support for the expansion of SIFs and since introduced new legislation repealing some of the more burdensome requirements of the previous legislation, which has prompted cities across Canada to more aggressively pursue establishing SIFs [46]. However, under this new legislation, the federal government will maintain responsibility for the approval of new facilities and there remains a need to demonstrate a lack of impact on crime [19], thus continuing to subject the expansion of these critical health services to political processes.

It is unclear why so many bureaucratic requirements (e.g., police approval) must still be met to implement SIFs, and why health officials cannot simply implement SIFs where there is a demonstrated need without obtaining support from other stakeholders [28]. This in part reflects a longstanding over-emphasis on enforcement-based approaches despite pronouncements by federal politicians that drug use should be regarded first and foremost as a health issue [81, 82]. The internal inconsistency between such official statements, policies, and actions represent an unfortunate and unnecessary barrier to the expansion of SIFs as Canada grapples with an opioid overdose crisis. Although British Columbia has rapidly implemented low-threshold SIFs under the label of “overdose prevention sites” in response to a public health emergency [73], these actions could be interpreted to contravene federal drug laws. Specifically, an exemption from the federal Minister of Health is required to operate a health service where people consume illicit drugs. Further legislative changes are needed to address the internal inconsistencies of the current legislation and more fully equip health officials with the tools to rapidly implement and scale-up supervised injection facilities in response to localized overdose and infectious disease outbreaks.

Along with changes to approval processes under federal legislation, there remains a need to revisit the operating procedures of supervised injection facilities to ensure their optimization for injection drug-using populations, particularly highly vulnerable sub-populations. Most notably, under the parameters of federal drug laws and guidelines, sanctioned SIFs are unable to accommodate people who require manual assistance with injections. Previous research has shown that up to one third of PWID in Vancouver report requiring assistance with injections, including a disproportionate number of women [83], and that requiring assistance with injections increases vulnerability to HIV infection, overdose, and violence [84–86]. However, as demonstrated by the peer-run and unsanctioned SIF operated by VANDU, assisted injections administered in a regulated setting and in accordance with harm reduction practices can reduce these risks [40]. A further opportunity to align SIFs with the needs of drug-using populations would involve the addition of safer smoking rooms (SSRs) so as to accommodate individuals who inhale drugs, such as crack, methamphetamine, and heroin. While these interventions exist in some European settings and have been well accepted by those who inhale drugs, there are no sanctioned SSRs in Canada at this time [87], despite past feasibility research showing that a majority of crack smokers would be willing to use them [88]. Reforms are urgently needed to facilitate the integration of assisted injection and safer smoking interventions into SIFs and reduce challenges in access to these facilities stemming from gender, disability, and polysubstance use.

Furthermore, current gaps in coverage of supervised injection facilities point to the need to extend this evidence-based intervention into new settings and consider new approaches. For example, there is a growing body of literature regarding the challenges associated with in-hospital drug use, as well as current abstinence-focused hospital policies in driving discharges from hospital against medical advice [89]. Previous feasibility research has demonstrated a high willingness to use hospital-based supervised injection facilities among PWID [90], while qualitative research in a 24-h palliative and supportive care program with supervised consumption services demonstrated how this approach improves retention in care and minimizes drug-related risks [91]. While Edmonton has signaled its intention to open a hospital-based SIF, further steps are needed in other cities to extend this programming into hospital settings [55]. To more fully respond to the opioid overdose crisis, there is cause to further explore integrating supervised injection facilities into other settings where PWID commonly use drugs and experience overdoses and other adverse outcomes (e.g., emergency and social housing) and complementing fixed-site SIFs with mobile services to expand geographic coverage and ensure responsiveness to changing drug scene dynamics.

Finally, while SIFs have been primarily advanced as a health care service in Canada, the successes of peer-run SIFs point to the need to consider de-medicalizing these interventions through direct support for peer-based models. Although several peer-run overdose prevention sites are currently operating in British Columbia as part of the province’s response to the overdose crisis, federal regulations currently prohibit the establishment of peer-run SIFs [40]. The assumption that SIFs must be operated by health care professionals is at odds with previous studies demonstrating the feasibility and acceptability of peer-run supervised consumption services, and their role in reducing drug-related risks and harms. Not only have PWID in some cases expressed a strong preference for peer-run SIFs [92], but these have been also found to be uniquely positioned to extend coverage by engaging those who encounter social-structural barriers to accessing sanctioned interventions [40]. Building on the successes of these approaches represents one of the most promising ways to harness peer networks and community expertise to respond to the opioid crisis. To facilitate the creation and continued functioning of peer-run SIFs, amendments to federal laws should be made to allow PWID to work in SIFs. Further, local health authorities should seek to promote the operation of peer-run SIFs and provide necessary financial support given existing evidence indicating that peer-run SIFs extend the reach and coverage of these programs [40].

## Conclusions

In conclusion, our review of Canada’s experience with SIFs demonstrates that although considerable progress has been made towards integrating this form of intervention into the continuum of programs offered to PWID, continued activism, research advocacy, and litigation has been necessary in order to advance this evidence-based approach in Canada. Presently, increased acceptance of SIFs as a result of Canada’s overdose crisis and political changes has led to the rapid escalation of efforts to expand SIFs across the country. Notwithstanding the importance of these developments, there remains a pressing need to amend federal legislation to better enable the scale-up of these services. Although in many settings, such as Vancouver, access to a range of services such as SIF and naloxone distribution has increased [93], the ongoing overdose crisis indicates clearly that more must be done [69]. Further, models that are more responsive to the needs of PWUD (e.g., assisted injection services, peer-run models) should be implemented and evaluated, and SIF programming should be extended into new settings (e.g., hospital). Only then will Canada be truly maximizing the many opportunities for supervised injection facilities to reduce harm and health inequities.

## Abbreviations

DTES: Downtown Eastside
OPS: Overdose prevention sites
PHS: Portland Hotel Society
PWID: People who inject drugs
RNABC: Registered Nursing Association of British Columbia
SIFs: Supervised injection facilities
SSRs: Safer smoking rooms
VANDU: Vancouver Area Network of Drug Users
